# A Network of SCOP Hidden Markov Models and Its Analysis

**DOI:** 10.1186/1471-2105-12-191

**Published:** 2011-05-23

**Authors:** Liqing Zhang, Layne T Watson, Lenwood S Heath

**Affiliations:** 1 Department of Computer Science, Virginia Tech, Blacksburg, VA 24061, USA; 2 Department of Mathematics, Virginia Tech, Blacksburg, VA 24061, USA

## Abstract

**Background:**

The Structural Classification of Proteins (SCOP) database uses a large number of hidden Markov models (HMMs) to represent families and superfamilies composed of proteins that presumably share the same evolutionary origin. However, how the HMMs are related to one another has not been examined before.

**Results:**

In this work, taking into account the processes used to build the HMMs, we propose a working hypothesis to examine the relationships between HMMs and the families and superfamilies that they represent. Specifically, we perform an all-against-all HMM comparison using the HHsearch program (similar to BLAST) and construct a network where the nodes are HMMs and the edges connect similar HMMs. We hypothesize that the HMMs in a connected component belong to the same family or superfamily more often than expected under a random network connection model. Results show a pattern consistent with this working hypothesis. Moreover, the HMM network possesses features distinctly different from the previously documented biological networks, exemplified by the exceptionally high clustering coefficient and the large number of connected components.

**Conclusions:**

The current finding may provide guidance in devising computational methods to reduce the degree of overlaps between the HMMs representing the same superfamilies, which may in turn enable more efficient large-scale sequence searches against the database of HMMs.

## Background

The Structural Classification of Proteins (SCOP) database is a comprehensive protein database that organizes and classifies proteins based on their evolutionary and structural relationships [1-3]. It is organized into four hierarchical levels: family, superfamily, fold, and classes. At the lowest level (family), individual proteins are clustered into families based on some criteria that may indicate their common evolutionary origin, such as having a pairwise sequence similarity of more than 30% or lower sequence similarity but similar functions and structures. A good example of the latter is seen in globin proteins whose pairwise sequence similarities are much lower than 30% but which have similar protein functions. Next, families are grouped into superfamilies if their structures and/or function features indicate a possible common evolutionary origin. Then superfamilies are clustered into folds if superfamilies share major secondary structures with the same topological arrangements. Finally, different folds are grouped into classes based on their secondary structural compositions. Unlike the other levels, a class might not necessarily imply common evolutionary origins and exists more for convenience than for actual biological implications.

Apart from the hierarchical classification and organization of proteins, the SCOP database employs hidden Markov models (HMMs) to represent superfamilies [4,5]. The basic procedure of building an HMM for a particular superfamily starts with a seed protein and performs sequence search in a database to obtain other proteins that have sequence similarities above a set threshold. The newly obtained sequences are used to iterate the search for some number of times to obtain additional proteins. Finally, all sequences are aligned and an HMM is constructed for the multiple sequence alignment [4,5]. It has been shown that different seed proteins might produce HMMs that cover different members of the superfamily [4,5]. Thus, in order to represent the full set of proteins in a superfamily, multiple HMMs are built for the superfamily using multiple seed proteins. For example, the beta-beta-alpha zinc fingers superfamily has altogether 91 HMMs representing it, and the P-loop containing nucleoside triphosphate hydrolases superfamily has 406 HMMs representing it.

Because each superfamily might be represented by multiple HMMs, there may be a high degree of overlap and redundancy among the models. However, there have not been any studies examining this issue systematically. To understand how the HMMs in the SCOP database are related to one another and the degree of overlap or redundancy among HMMs from either the same or different superfamilies, we perform a detailed analysis of the HMMs in SCOP for their similarity and relationships using a network approach. Specifically, we perform an all-against-all HHsearch for the library of HMMs in the SCOP database.

HHsearch is similar to BLAST, except that instead of matching a sequence against a database of sequences, it uses a query HMM or sequence to match against a database of HMMs and identifies the HMMs significantly homologous to the query HMM or sequence [6]. We then construct a network of HMMs, where the link between two HMMs is based on their similarity, and examine some commonly evaluated network properties. We compare the current network with previously documented networks and outline some questions for future research.

## Results and Discussion

### General statistics of the HMMs and their network

A general description of the HMMs used to construct the network is shown in Table 1. There are seven classes in the collection of HMMs, falling into 721 folds, 1163 superfamilies, and 2573 families. Class *c *has the highest number of HMMs (3391) and class *f *the fewest (145).

**Table 1:** The general statistics of the HMM library

Class	Number of HMMs	Number of folds	Number of superfamilies	Number of families
a	1975	157	262	506
b	2590	109	231	485
c	3391	120	194	686
d	2932	223	328	683
e	199	34	34	51
f	145	29	44	50
g	697	49	70	112
All	11929	721	1163	2573

**Figure 1:**
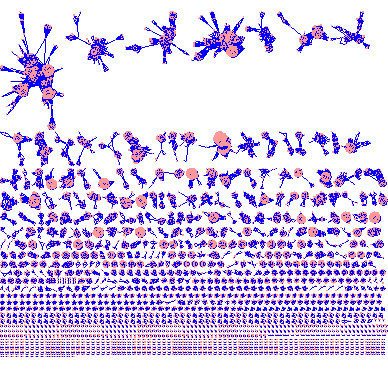
**The HMM network**.

**Figure 2:**
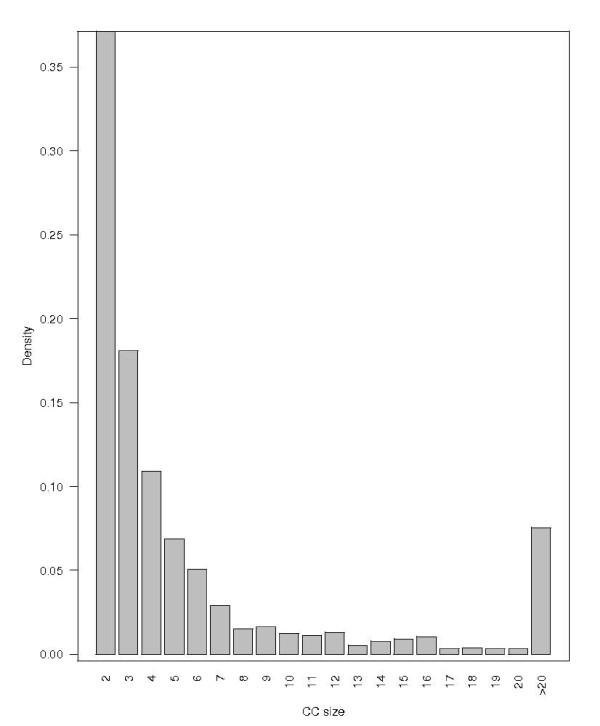
**Size distribution of connected components**. The CC size ranges from 2 to 590, with median 3 and mean 7.8.

The entire HMM network is shown in Figure 1, where the e-value cutoff is 0.001. There are altogether 151,461 edges for the 11,929 vertices. A significant property shown in Figure 1 is that the entire network is highly disconnected, with many much smaller connected components. In fact, there are altogether 1524 connected components (CCs). The size distribution of CCs is shown in Figure 2. The smallest CC contains two vertices, the largest 590 vertices, 566/1524 = 37% contain only two vertices and about 73% contain five or fewer vertices. The median CC size is 3 and the mean 7.8. The top 20 largest CCs are listed in Table 2.

**Table 2:** The 20 largest connected components and their densities

Size rank	Number of vertices	Density
1	590	0.12
2	349	0.21
3	277	0.65
4	155	0.15
5	141	0.38
6	121	0.33
7	120	0.19
8	106	0.72
9	99	0.84
10	90	0.95
11	86	0.99
12	85	0.89
13	81	0.32
14	80	0.83
15	74	0.66
16	73	0.65
17	72	0.16
18	70	1.00
19	69	0.97
20	66	0.40
All	11929	0.002

### Degree distribution

**Figure 3:**
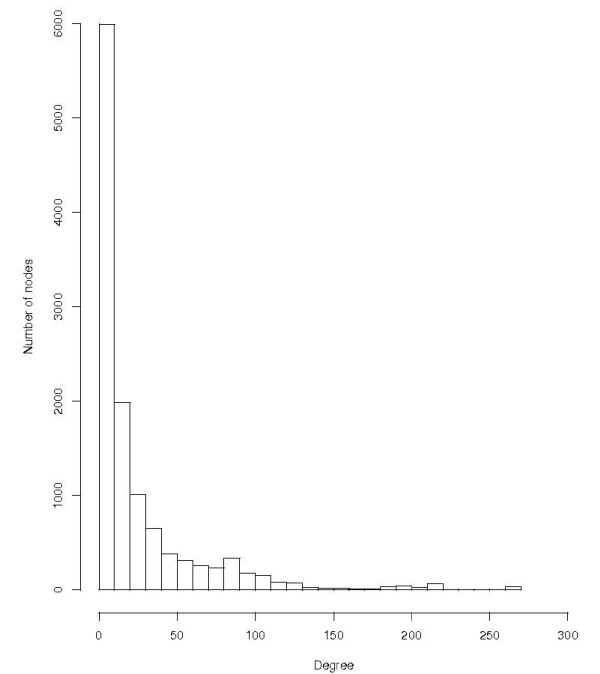
**The distribution of the degrees of the HMM network**.

**Figure 4:**
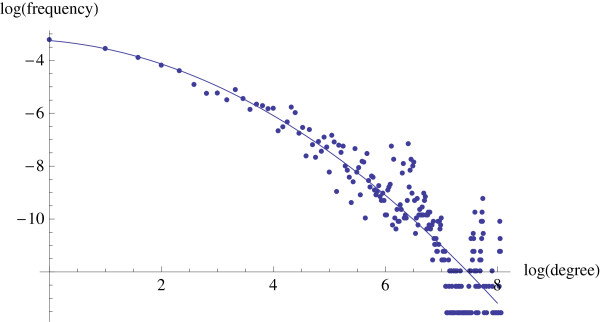
**Log-log degree distribution**. The log base is 2. The best fitting quadratic curve is 3.2481 - 0.176557*x *- 0.133088*x*^2^.

The distribution of the degrees of the HMM network is shown in Figure 3. Degree ranges from 1 to 268, with the average of 26 and median of 10. The log-log degree distribution is also shown (Figure 4). It is evident that a power law distribution does not fit the data. The best fitting quadratic curve is also plotted with the data. It provides a relatively good fit for the smaller values of log(degree), and then towards the larger degrees, the fit is not so good.

### Network Density

**Figure 5:**
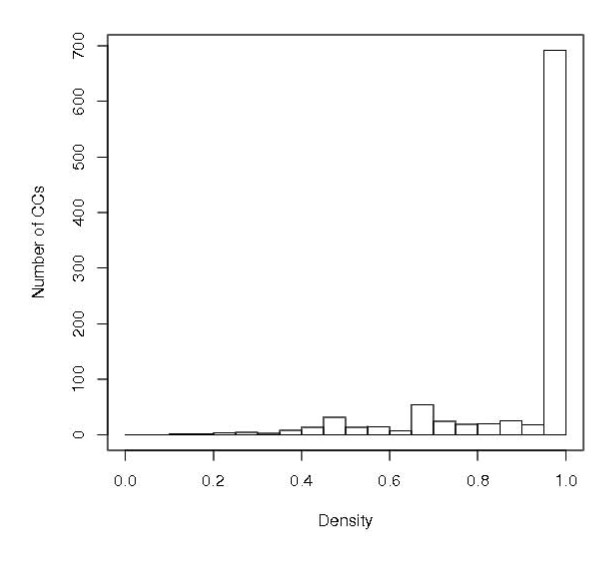
**The density distribution of CCs**. CCs with size two are excluded from the distribution.

Density, computed as the number of edges over the number of all possible edges (in a fully connected graph), provides some quantitative evaluation on the connectivity of a network. The density of the entire network is low, only 0.002 . In contrast, individual CCs tend to have high densities (Figure 5), with more than 82.5% of CCs having density greater than 0.95. 1236 CCs are fully connected, i.e., cliques, with the largest clique of size 70. Overall, 566 have size 2, 261 size 3, 140 size 4, 85 size 5, 60 size 6, and 124 sizes greater than 6.

Thus, individual CCs tend to have very high connectivity, whereas the entire network is not well connected. The density of the 20 largest CCs is shown in Table 2. The largest CC with 590 vertices has the lowest density, and the 18th largest CC with 70 vertices has a density of 1, and is therefore a fully connected component. There is a significant negative correlation between CC size and density (Kendall's rank correlation *τ *= -0.43, *p*-value < 2.2 · 10^-16 ^for CC size > 2).

### Vertex centrality

Vertex centrality measures the "importance" of a vertex. Two centrality metrics, degree and betweenness, were computed for the vertices in the entire HMM network. The top 20 HMMs that have the highest degrees all belong to the same superfamily, b.1.1, Immunoglobulin, and also to the third largest CC that has 277 vertices. Thus, these 20 HMMs are connected with almost all other HMMs in the third CC. The HMM d1n26a1 (SCOP ID b.1.1.4, (A:1-93)) has the highest degree, 268, belonging to the Interleukin-6 receptor alpha chain, N-terminal domain. Table 3 shows the top 20 HMMs that have the highest betweenness. Thirteen of the 20 HMMs belong to the superfamily c.2.1 (NAD(P)-binding Rossmann-fold domains), two to the superfamily b35.1.2, and two to the superfamily c.37.1. Eighteen of the 20 HMMs belong to the largest CC and the two remaining (c.37.1.14 and c.37.1.11) to the second largest. The HMM d1bg6a2 (SCOP ID c.2.1.6, (A:4-187)) has the highest betweenness, 14916, belonging to N-(1-D-carboxylethyl)-L-norvaline dehydrogenase (Arthrobacter, strain 1c). Interestingly, there is no overlap of HMMs that have the highest of both degree and betweenness.

**Table 3:** The 20 HMMs with largest betweenness

Rank	HMM ID	SCOP ID	Betweenness
1	d1bg6a2	c.2.1.6	14915.8
2	d1o8ca2	c.2.1.1	14665.7
3	d1e5qa1	c.2.1.3	14504.0
4	d2bzga1	c.66.1.36	9557.9
5	d3bswa1	b.81.1.8	9168.0
6	d1vj0a2	c.2.1.1	8211.0
7	d1ks9a2	c.2.1.6	7469.9
8	d2bmfa2	c.37.1.14	7439.8
9	d2dt5a2	c.2.1.12	7410.7
10	d1pjca1	c.2.1.4	7325.1
11	d1gtea4	c.4.1.1	7165.3
12	d1gu7a1	b.35.1.2	6768.0
13	d1tt7a1	b.35.1.2	6768.0
14	d2f1ka2	c.2.1.6	5985.2
15	d1ebfa1	c.2.1.3	5959.8
16	d1jqba2	c.2.1.1	5313.1
17	d1gr0a1	c.2.1.3	5220.0
18	d1ye8a1	c.37.1.11	5207.7
19	d1piwa2	c.2.1.1	4556.8
20	d1hdoa_	c.2.1.2	4403.8

Because the entire HMM network contains many CCs, among which there are no connections, we computed three centrality measurements (degree, betweenness, and closeness) for the 20 largest CCs. Table 4 lists the two HMMs that have one of the highest centrality measurements. Unlike the observation for the entire network, there is great consistency between HMMs with the highest three centrality measurements, i.e., the same HMMs that have one of the highest centrality measurements, tend to also have the highest other two measurements. For example, for the largest CC, d1bg6a2 is the HMM with the highest betweenness, closeness, and the second highest degree. Because the network has many connected components, examining the importance of vertices for these subnetworks seems to be more meaningful than for the entire network. It is thus very useful to observe that different measurements of vertex centrality give similar results, suggesting that one does not have to be overly concerned with the choice of centrality measurements when determining the important HMMs.

**Table 4:** The top 2 HMMs with the highest centrality measurements for the 20 largest CCs.

CC	HMM	B	HMM	C	HMM	D
1	d1bg6a2 (c.2.1.6)	14915.8	d1bg6a2 (c.2.1.6)	0.51	d1e5qa1 (c.2.1.3)	222
1	d1o8ca2 (c.2.1.1)	14665.7	d1e5qa1 (c.2.1.3)	0.50	d1bg6a2 (c.2.1.6)	183
2	d2bmfa2 (c.37.1.14)	7439.8	d1ye8a1 (c.37.1.11)	0.69	d1ye8a1 (c.37.1.11)	219
2	d1ye8a1 (c.37.1.11)	5207.7	d2i3ba1 (c.37.1.11)	0.65	d1bifa1 (c.37.1.7)	206
3	d1gsma1 (b.1.1.4)	546.2	d1n26a1 (b.1.1.4)	0.97	d1n26a1 (b.1.1.4)	268
3	d1l6za2 (b.1.1.4)	514.7	d1f2qa1 (b.1.1.4)	0.96	d1f2qa1 (b.1.1.4)	265
4	d1tqja_ (c.1.2.2)	1931.5	d1yxya1 (c.1.2.5)	0.56	d1y0ea_ (c.1.2.5)	71
4	d1izca_ (c.1.12.5)	1712.9	d1y0ea (c.1.2.5)	0.56	d1gtea2 (c.1.4.1)	68
5	d1wjka_ (c.47.1.1)	1042.1	d1a8la2 (c.47.1.2)	0.69	d1a8la2 (c.47.1.2)	88
5	d1r7ha_ (c.47.1.1)	683.6	d1f9ma (c.47.1.1)	0.69	d1ep7a_ (c.47.1.1)	87
6	d1gjwa2 (c.1.8.1)	1318.6	d1ecea (c.1.8.3)	0.65	d1ecea_ (c.1.8.3)	76
6	d1bf2a1 (b.1.18.2)	1199.0	d1qnra (c.1.8.3)	0.61	d1qnra_ (c.1.8.3)	75
7	d1jhfa1 (a.4.5.2)	2369.2	d2d1ha1 (a.4.5.50)	0.53	d1ub9a_ (a.4.5.28)	58
7	d1fsea_ (a.4.6.2)	1988.3	d1sfxa (a.4.5.50)	0.52	d2d1ha1 (a.4.5.50)	55
8	d1tcaa_ (c.69.1.17)	390.0	d1tcaa (c.69.1.17)	0.93	d1tcaa_ (c.69.1.17)	97
8	d1ispa_ (c.69.1.18)	167.6	d1b6ga (c.69.1.8)	0.92	d1b6ga_ (c.69.1.8)	96
9	d1cd9b1 (b.1.2.1)	224.1	d1bqua1 (b.1.2.1)	0.95	d1cd9b1 (b.1.2.1)	95
9	d2c4fu1 (b.1.2.1)	193.0	d1cd9b1 (b.1.2.1)	0.95	d1bqua1 (b.1.2.1)	93
10	d1wg4a_ (d.58.7.1)	14.8	d1wg4a (d.58.7.1)	1.00	d1wg4a_ (d.58.7.1)	89
10	d1whya_ (d.58.7.1)	13.8	d1fxla1 (d.58.7.1)	0.99	d1fxla1 (d.58.7.1)	88
11	d1p3wa_ (c.67.1.3)	0.4	d1p3wa (c.67.1.3)	1.00	d1p3wa_ (c.67.1.3)	85
11	d1fg7a_ (c.67.1.1)	0.4	d1fg7a (c.67.1.1)	1.00	d1fg7a_ (c.67.1.1)	85
12	d1tiza_ (a.39.1.5)	175.3	d1tiza (a.39.1.5)	0.98	d1tiza_ (a.39.1.5)	82
12	d1 fi5a_ (a.39.1.5)	68.1	d1 5a (a.39.1.5)	0.97	d1rroa_ (a.39.1.4)	81
13	d1onwa1 (b.92.1.7)	362.1	d1ra0a2 (c.1.9.5)	0.66	d1ra0a2 (c.1.9.5)	42
13	d2bb0a1 (b.92.1.10)	252.0	d1nfga2 (c.1.9.6)	0.64	d1i0da_ (c.1.9.3)	41
14	d1agja_ (b.47.1.1)	132.4	d1agja (b.47.1.1)	0.98	d1agja_ (b.47.1.1)	77
14	d1l1ja_ (b.47.1.1)	132.4	d1l1ja (b.47.1.1)	0.98	d1l1ja_ (b.47.1.1)	77
15	d1yvka1 (d.108.1.1)	225.4	d1wwza1 (d.108.1.1)	0.85	d1wwza1 (d.108.1.1)	63
15	d1vhsa_ (d.108.1.1)	148.8	d1bo4a (d.108.1.1)	0.85	d1bo4a_ (d.108.1.1)	63
16	d1qhqa_ (b.6.1.1)	74.3	d1e30a (b.6.1.1)	0.94	d1e30a_ (b.6.1.1)	67
16	d1e30a_ (b.6.1.1)	65.1	d1kcwa2 (b.6.1.3)	0.90	d1kcwa2 (b.6.1.3)	64
17	d1huxa_ (c.55.1.5)	1183.4	d1huxa (c.55.1.5)	0.63	d1huxa_ (c.55.1.5)	38
17	d2ch5a1 (c.55.1.5)	341.4	d2ewsa1 (c.55.1.14)	0.54	d2ewsa1 (c.55.1.14)	28
18	d1rgwa_ (b.36.1.1)	0.0	d1rgwa (b.36.1.1)	1.00	d1rgwa_ (b.36.1.1)	69
18	d1t2ma1 (b.36.1.1)	0.0	d1t2ma1 (b.36.1.1)	1.00	d1t2ma1 (b.36.1.1)	69
19	d1j7la_ (d.144.1.6)	2.5	d1j7la (d.144.1.6)	1.00	d1j7la_ (d.144.1.6)	68
19	d1zara2 (d.144.1.9)	2.5	d1zara2 (d.144.1.9)	1.00	d1zara2 (d.144.1.9)	68
20	d2fug34 (d.58.1.5)	1050.3	d2fug34 (d.58.1.5)	0.63	d2fdna_ (d.58.1.1)	32
20	d3c8ya2 (d.15.4.2)	1045.0	d3c8ya2 (d.15.4.2)	0.59	d7fd1a_ (d.58.1.2)	32

For each row, the columns refer to the rank of the CC based on its size, the HMMs (SCOP IDs in the parenthesis) with the largest or second largest centrality measured by betweenness (B), closeness (C), and degree (D).

The results show that from the entire network, the vertices with the highest degrees do not necessarily have the highest betweenness, and vice versa. Degree measures how many immediate neighbors one HMM has, and therefore, the more it has, the more central it is. The vertices with the 20 largest degrees are all from the third largest CC, and are connected to about 94% of its vertices. The vertices with the 20 largest betweenness are from either the largest CC or the second largest CC. Since betweenness reflects how essential one vertex is to the connection of any other two vertices in the graph, in the case of HMMs, it may reflect the possibility that one HMM is the *hybrid *of two HMMs, that is, between the two HMMs, there is no significant similarity, but through the one HMM, the HMMs can be linked. Biologically, this idea seems to reflect hybrid or mosaic proteins where one protein contains domains from multiple proteins. To our knowledge, the idea of hybrid HMMs has not been discussed previously and deserves more research attention. Moreover, we hypothesize that the HMMs with high centrality measurements may be better able to pick up the sequences that belong to the superfamily than the more peripheral HMMs. This idea seems to be especially promising considering the observation that the three centrality measurements identify similar sets of vertices within the connected components. Future studies can be directed to test this hypothesis.

### Network diameter

The diameter of the largest CC (containing 590 vertices) is 9. The average distance between the vertices in the component is 2.94. This bears some similarity to the yeast protein interaction network [7], constructed using the protein interaction data from the January 2007 version of the BioGRID database, an online repository for interaction datasets aggregated from both high-throughput data and focused individual studies for the affinity of interacting protein pairs [8,9]. This protein interaction network consists of 5,151 proteins and 31,201 interactions. Its largest CC (containing 5,128 vertices) also has the same diameter of 9, but a larger average distance of 3.68. Thus, this protein interaction network seems to have more vertices that are a bit more spread out, which contributes to a larger average distance. To this point, it is very interesting that despite the big difference in the sizes of the two CCs of the two networks, the diameters are the same.

**Figure 6:**
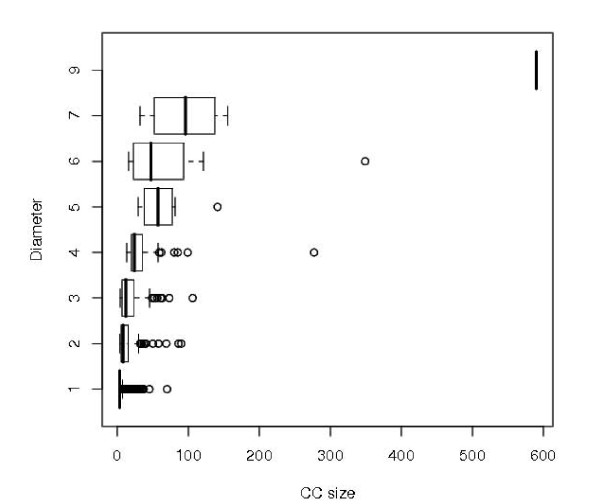
**Boxplot for the diameter of CCs as a function of CC size**. The box marks the lower and upper quantile of CC sizes with the same diameter, the dark line marks the median, the whiskers mark the border of lower and upper outliers with the dots outside denoting the outliers.

We also measured the diameters of all the CCs to see how they change as a function of CC size. Figure 6 shows that larger CCs tend to have larger diameters. However, smaller CCs can have large diameters as well. For example, a CC of size 32 has diameter seven, the same as a CC of size 155; a CC size of 16 has diameter six, the same as a CC of size 121. There are 1236 CCs with diameter 1, corresponding to the number of cliques.

### The effect of e-value cutoff on the network

**Figure 7:**
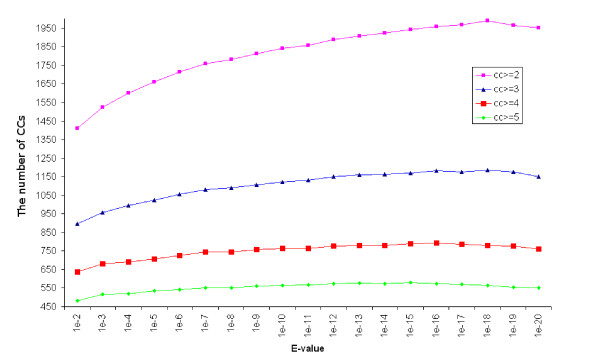
**The number of CCs of size > 1 as a function of e-value cutoff**.

**Figure 8:**
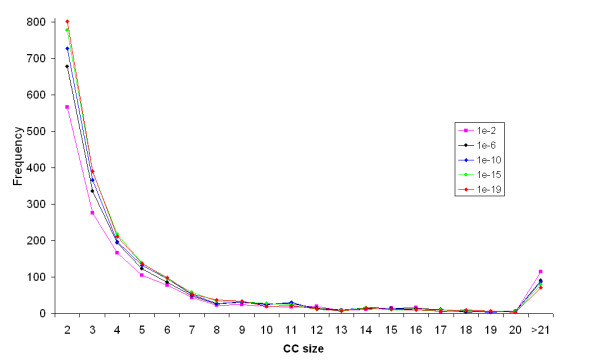
**CC size distribution as a function of e-value cutoff**. For clarity, only the distributions for some e-value cutoffs from 10^-20 ^to 10^-3 ^are shown.

As the e-value measures the degree of similarity between two HMMs, we examined how changing e-value cutoff affects the general properties of the network, such as the number and sizes of CCs. Figure 7 shows the effect of changing the e-value cutoff on the number of CCs in the network. The number of CCs increases gradually with the more stringent e-value cutoffs, reaching the highest for 10^-18 ^(the slight drop for e-value cutoffs of 10^-19 ^and 10^-20 ^is due to the exclusion of CCs of size 1). Similar patterns are observed when only CCs that are greater than size two, three, and four are considered, generally, the number of CCs increases with more stringent e-value cutoffs. To see what specific sized CC groups are more affected by the stringency of e-value cutoffs, the CC size distribution was also studied as a function of e-value cutoffs. Figure 8 shows that changing e-value cutoff has the largest effect on the number of CCs that are of size two, and the effect reduces greatly for larger sized CCs. With low stringency e-value cutoffs, there are more larger CCs.

**Figure 9:**
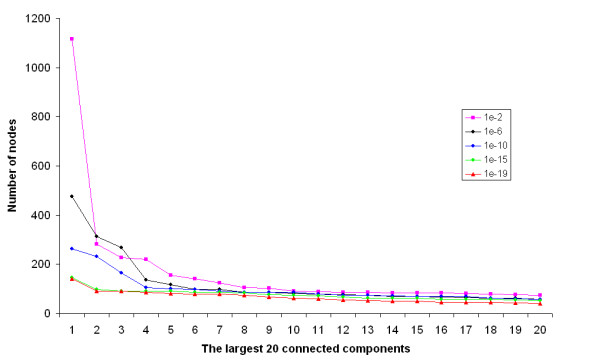
**The 20 largest connected components and e-value**. For clarity, only the curves for some e-value cutoffs from 10^-20 ^to 10^-3 ^are shown.

Figure 9 shows the sizes of the 20 largest CCs with varying e-value cutoffs. The e-value cutoff has a more pronounced effect on the sizes of the largest CCs than on those of the smaller CCs. For example, there are almost twice as many vertices in the largest CC for e-value cutoff of 0.01 as for 0.001. Thus the low e-value of 0.01 allows the formation of really large CCs that may include some low similarities between HMMs. The number of vertices contained in the same ranked CCs shows less difference after the second largest CC.

### CCs and SCOP hierarchy

Within the CCs, we examined whether the HMM members are from the same family, superfamily, fold, or class. There are altogether 1178 CCs whose members have the same SCOP domain classification (conserved at all hierarchical levels), 271 CCs whose HMMs belong to the same superfamily but to different families, 24 whose members belong to the same fold, but to different superfamilies, 18 whose members belong to the same class but have different folds, and the remaining 33 whose members are from different classes.

**Figure 10:**
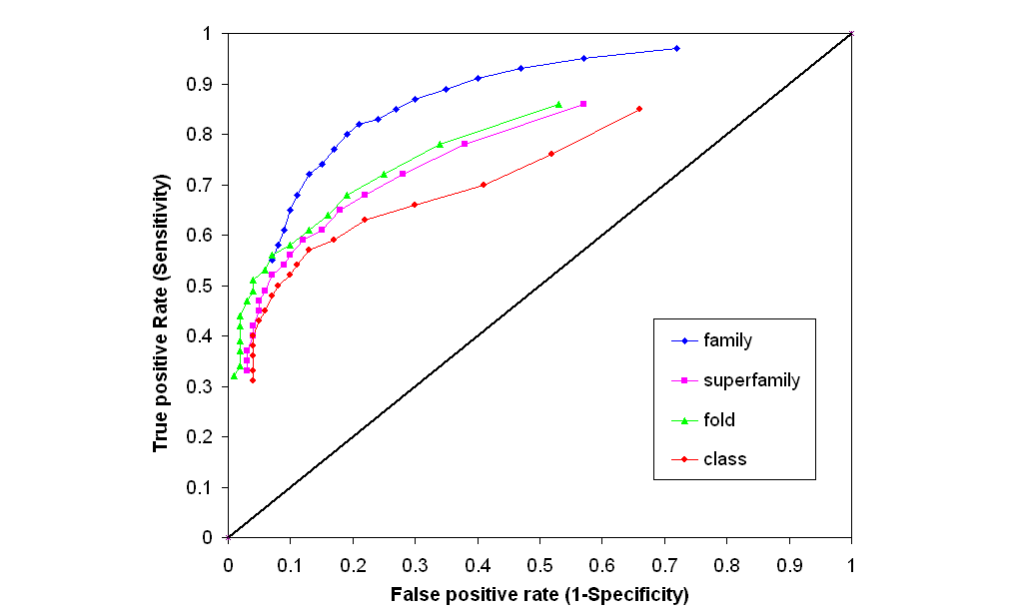
**The ROC curves**. The ROC curves for family, superfamily, fold, and class with different e-value cutoffs. For each curve, the data points from left to right correspond to the FPR and TPR for the e-value cutoffs from 10^-20 ^to 10^-3^.

The consistency between the prediction of HMM memberships at different hierarchical levels in the SCOP database based on the e-value cutoffs and the classification of the SCOP database was evaluated by ROC curves, shown in Figure 10. We make several observations. First, for all four levels of the hierarchy, the higher the e-value cutoff, the higher the sensitivity (true positive rate), so is the false positive rate, which is expected because higher e-value means a less stringent prediction criterion that in turn leads to a higher number of true positive predictions, and also a higher number of false positive predictions. Meanwhile, the rate of increase in sensitivity outpaces the rate of increase in the false negative rate as the e-value becomes more stringent, suggesting that beyond a certain e-value cutoff, the HMMs belonging to the same hierarchical levels also tend to have high similarity, which make them robust to the e-value cutoff change. Second, the curves for the prediction of fold and superfamily are very similar to each other, indicating that for the same e-value cutoff, the predictions for whether two HMMs belong to the same fold or superfamily are similar. In fact, for the same e-value cutoff, the difference in true positive rate (sensitivity) between the fold and superfamily ROC curves is either 0 or 0.01, and the difference in false positive rate (1-specificity) falls within the narrow range [0.01-0.04]. Third, the prediction quality is the worst for class as compared to the other three levels, with worst sensitivity and specificity for the same e-value cutoffs. This might not be so surprising as classification at the class level is more for convenience than for biological reasons.

Because fold and superfamily show similar classifications, we focused on studying the superfamilies further. In order to see how the superfamilies are represented in terms of connected components, we examined the number of HMMs representing the 1163 superfamilies to see how many CCs the HMMs are dispersed into. Table 5 shows the top ten superfamilies that have the highest number of HMM representations and also the top ten superfamilies that are split into the highest number of CCs. It shows that superfamilies differ in the extent of dispersion of their HMMs, with some superfamilies having really high dispersal, some very low. For example, the superfamily c.69.1 has 106 HMMs, all clustered into the same CC (CC size rank #8). In contrast, a.4.5 has 150 HMMs, but dispersed into 20 CCs. We also computed a dispersal index (the number of CCs/the number of HMMs) for all the superfamilies, and found that the superfamilies with the highest dispersal are dominated by superfamilies that have only one or two HMMs, and the superfamilies with a large number of HMMs tend to have low dispersal, in fact, among the 20 superfamilies with the lowest dispersal, six of them have the largest number of HMMs.

**Table 5:** Functional annotation of the top ten superfamilies that have either the largest number of HMM representations or CCs.

Superfamily ID	# of HMMs	# of CCs	Functional annotation
c.37.1	358	3	P-loop containing nucleoside triphosphate hydrolases
b.1.1	286	6	Immunoglobulin
c.2.1	267	2	NAD(P)-binding Rossmann-fold domains
a.4.5	150	20	Winged helix DNA-binding domain
c.47.1	147	4	Thioredoxin-like
c.1.8	141	7	(Trans)glycosidases
c.66.1	119	2	S-adenosyl-L-methionine-dependent methyltransferases
a.4.1	110	8	Homeodomain-like
c.69.1	106	1	alpha/beta-Hydrolases
b.1.2	98	2	Fibronectin type III

**Superfamily ID**	**# of CCs**	**# of HMMs**	**Functional annotation**
a.4.5	20	150	Winged helix DNA-binding domain
b.1.18	17	76	E set domains
b.40.4	16	95	Nucleic acid-binding proteins
b.29.1	14	97	Concanavalin A-like lectins/glucanases
d.14.1	11	52	Ribosomal protein S5 domain 2-like
g.39.1	10	83	Glucocorticoid receptor-like (DNA-binding domain)
b.18.1	10	54	Galactose-binding domain-like
d.3.1	10	54	Cysteine proteinases
a.4.1	8	110	Homeodomain-like
b.121.4	8	58	Positive stranded ssRNA viruses

### The working hypothesis

Taking into account the processes that built the HMMs and the hierarchical classification of the HMMs in the SCOP database, we hypothesize that the network should reflect this process, i.e., *the HMMs in a connected component belong to the same family or superfamily more often than expected under a random network connection model*. The results show strong evidence that HMMs in a connected component tend to represent the same family or superfamily. Among the total 1524 CCs, more than 77% have only members from the same family; more than 95% have only members from the same superfamily. Thus, there is overwhelming evidence supporting our working hypothesis that HMMs belonging to the same family or superfamilies tend to cluster together in the network.

However, to formally evaluate this and provide some statistical support, we also simulated 10,000 random networks, while preserving the degree distribution and the number and sizes of connected components. Each random network has the same number of connected components as our original network, and the working hypothesis predicts that the connected components of such a network have a lower degree of conservation in the family and superfamily assignment. Among the 10,000 simulated random networks, the highest proportions of CCs having only members from the same family and superfamily are as low as 0.5% and 0.7%. This shows that in the observed network, the HMMs from the same family or superfamily do have a strong tendency to cluster, agreeing with our working hypothesis.

### Comparison with other networks

It is evident that the HMM network is highly clustered. In fact, its clustering coefficient is 0.85, which, to our knowledge, seems to be the highest among the biological networks that have been studied so far. As shown by Newman [10], the undirected networks that tend to have high clustering coefficients are social networks. For example, the film directors network has a clustering coefficient of 0.20 and coauthorship networks for math, physics, and biology disciplines are 0.15, 0.45, and 0.088, respectively, whereas biological networks such as metabolic network and protein interaction network have only a clustering coefficient of 0.09 and 0.07, respectively [10]. The comparison indicates that the current network has distinct features from the previously characterized real-world networks.

## Conclusions

In this paper, we examined the properties of the network constructed for HMM models in the SCOP protein structural classification database. A number of questions remain to be addressed in future research. For example, can we devise a computational method to measure or evaluate the degree of redundancy or overlap between HMM models that are used to represent the same superfamily? This research is meaningful given the ever increasing number of large-scale genomic sequences (therefore more protein sequences). Given that we can measure the redundancy of the HMMs of a superfamily, the logical question becomes, can we computationally reduce the redundancy of the HMM library, e.g., possibly by constructing super-HMMs, each of which represents a collection of redundant HMMs, so that a protein sequence is scanned against a reduced set of HMMs (super-HMMs) rather than the entire set of HMMs that have overlaps and redundancies? Finally, because the HMM network shows distinct properties from many documented networks as discussed above, can we propose a theoretical model to better account for the observations in the current network? Moreover, as our HMM network is also weighted, with edges quantifying the similarity between two HMMs, future proposed models can also consider the incorporation of weighted edges into the network.

## Methods

The SCOP library of HMMs (scop70_1.75.hhm.tar.gz) was downloaded from the website http://scop.mrc-lmb.cam.ac.uk/scop/count.html#scop-1.75, where the SCOP version was filtered to 70% maximum pairwise sequence identity. The library contains a total of 13,730 HMMs, from seven classes *a, b, c, d, e, f, g*, where class *a *contains only *α *(i.e., *α *helix) proteins, class *b *contains only *β *(i.e., *β *sheet) proteins, class *c *contains *α *and *β *proteins (mainly parallel *β *sheets (*beta - alpha - beta *units)), class *d *contains *α *and *β *proteins (mainly antiparallel *β *sheets, i.e., segregated *α *and *β *regions), class *e *contains multi-domain proteins (i.e., folds consisting of two or more domains belonging to different classes), class *f *contains membrane and cell surface proteins, and class *g *contains small proteins. It is useful to mention that the SCOP domain classification ID specifies the entire hierarchy, e.g. c.1.1.1, the first field is for the class *c*, second for the fold, third for the superfamily, and the last for the family.

HHsearch [6] was performed for all-against-all HMMs with the default parameters. The command used was "hhsearch -i hmm.hhm -d hmmlib.hhm", where hmm.hhm is the query HMM and hmmlib.hhm is the library of all the HMMs. The secondary structure scoring option was not used, as our goal was not to detect remote homology between HMMs and sequences. According to the HHsearch authors, no calibration is necessary, as the HHsearch is performed on the SCOP database. HHsearch, similar to BLAST, uses a query that can be either a protein sequence or an HMM to search a database of sequences or HMMs and identify homology between the query and sequences and HMM models in the databases that is above a given threshold. In the current study, the e-value, a measurement of homology similar to BLAST's e-value, was set to 0.001. This e-value cutoff has also been used by Pfam to identify a Pfam clan [11], which is essentially equivalent to the superfamily hierarchy. A total of 13,547 HMMs have matches that met the criterion, with 1,618 having no other matches except themselves. Thus, 11,929 HMMs were used for the subsequent network analysis.

To study the relationship of the HMMs, an undirected network *G *= (*V*, *E*) was constructed, where the vertices *V *are HMMs, and there is an edge in *E *between two HMMs if their e-value is below the threshold. General network statistics were computed, and a quadratic function was fitted to the log-log degree distribution. Three common vertex centrality measurements, degree centrality, betweenness centrality, and closeness centrality, were computed to evaluate the "importance" of vertices in the network. The degree of a vertex *a *is the number of edges incident on *a*. Betweenness for a vertex *a*,(1)

introduced in Freeman [12], measures roughly the number of shortest paths going through *a σ *(*s*, *t*) is the number of shortest paths between vertices *s *and *t*, and *σ *(*s*, *t *| *a*) is the number of shortest paths between vertices *s *and *t *that go through *a*. Thus, the higher the betweenness of a vertex, the more "central"/important the vertex is. In a fully connected network, the betweenness of all vertices is 0.

The closeness centrality measures the number of steps required to access every other vertex from a given vertex, specifically, the closeness of a vertex *a*, *c *(*a*), is computed by(2)

where *d_a, i _*is the length of the shortest path between vertex *a *and vertex *i*. Closeness ranges from 0 (does not reach 0) to 1; the higher it is for a vertex, the more "central" the vertex is. These centrality measurements have different motivations and show different aspects for the importance of vertices in a network.

The network clustering coefficient, C, also known as transitivity, measured by the ratio between the number of triangles and the number of connected triplets, was computed for the entire network. The number of connected components that are trees, where there are *N *vertices but only *N *- 1 edges between the vertices, was computed for the entire network as well.

To systematically study the consistency between the e-value cutoffs for the prediction of whether or not HMMs belong to the same hierarchical level and classification of the SCOP database, we examined the Receiver Operating Characteristic (ROC) curves for the prediction of the hierarchical categories of two HMMs provided by different e-value cutoffs. The ROC curve shows how the true positive rate changes with the false positive rate for a classification. Specifically, for example, at the family level, if a sample of two HMMs were classified to the same family by the SCOP database, the prediction based on a specific e-value cutoff is considered to be a false negative (FN) if the e-value similarity of the two HMMs is worse/higher than the e-value cutoff, a true positive (TP) if the e-value is better (i.e., lower) than the cutoff, if the two HMMs were not classified to the same family by the SCOP database, the prediction based on the specific e-value cutoff is considered to be a true negative (TN) if the e-value similarity of the two HMMs is worse/higher than the e-value cutoff, a false positive (FP) if their e-value is better (i.e., lower) than the cutoff. Similar rules were applied to classify each pair of HMMs into the four categories (TP, FP, FN, and TN), for the four hierarchies, class, fold, superfamily, and family. True positive rate (i.e., sensitivity) was calculated as(3)

and false positive rate (ie., 1 - specificity) as(4)

An ROC curve was plotted for the four levels (i.e., class, fold, superfamily, and family) with different e-value cutoffs ranging from 10^-20 ^to 10^-3^.

## Authors' contributions

LZ, LTW, LSH conceived the research. LZ analyzed the data and wrote the manuscript. LZ, LTW, LSH revised the manuscript. All authors read and approved the final manuscript.
